# Influence of Different Fermentation Conditions on the Aroma-Active Compounds During New-Make Whisky Production Determined by GC-MS, GC×GC-O-MS, HPLC, and UPLC-MS

**DOI:** 10.3390/molecules31122138

**Published:** 2026-06-17

**Authors:** Xiaoduo Ma, Lei Xing, Ranran Feng, Shumin Hu, Wei Yong, Tianyang Guo, Zhaoxia Yang, Huanlu Song

**Affiliations:** 1 Tsingtao Brewery Co., Ltd., Qingdao 266061, China; mxd200104012@163.com (X.M.); xinglei@tsingtao.com.cn (L.X.); husm@tsingtao.com.cn (S.H.); 2 School of Food and Health, Beijing Technology and Business University, Beijing 100048, China; frr2801431930@126.com (R.F.); songhl@th.btbu.edu.cn (H.S.); 3 Institute of Food Safety, Chinese Academy of Quality and Inspection & Testing, Beijing 100176, China; benomyl@163.com

**Keywords:** new-make whisky, aroma compounds, fermentation, distillation, two-dimensional gas chromatography–olfactory–mass spectrometry

## Abstract

Whisky is well-known worldwide owing to its unique flavor, and its fermentation and distillation conditions have a significant effect on its aroma. In this paper, new-make spirits were prepared by four fermentation conditions (two distilling yeasts and two fermentation temperatures), and the different stages (wort, wash, low wine, and new-make spirit) of the samples were collected. The main aroma compounds and their precursors were initially determined in wort and wash samples by GC-MS, UPLC-MS and HPLC. Results showed that Strecker degradation, reduction, and esterification occurred during the fermentation process, leading to decreased contents of amino acids and increased contents of volatile esters, alcohols, and acids. Moreover, the two distilling yeasts exhibited their respective optimal fermentation temperatures. Then, the distillation process was evaluated by two-dimensional gas chromatography–olfactory–mass spectrometry (GC×GC-O-MS), and 74 aroma compounds were found in different stages of new-make whiskies fermented by the two distilling yeasts and one brewer’s yeast. The contents of most compounds were enhanced by at least ten times after two distillations, while the contents of some sulfur compounds decreased. Finally, the feature aroma-active compounds of each new-make whisky were identified according to rOAV. These results provided theoretical and methodological support for yeast selection and process control in whisky production.

## 1. Introduction

Whisky is a highly favored spirit around the world because of its strong and balanced aroma, and China is gradually becoming an important producer of whisky [1,2]. Interest in whisky production has shifted from alcohol yield to exploring how the process affects the flavor of whisky [3]. The quality of new-make whisky directly influences the final flavor of whisky, because its rich chemical components form the foundation for barrel aging. Apart from raw materials, the production process also has a significant impact on the quality of new-make whisky. This mainly includes fermentation and distillation, which respectively result from the action of microorganisms and heat-induced enrichment [4].

Yeasts not only convert sugar to alcohol, but also produce different aroma compounds, such as esters, alcohols, acids, aldehydes, and ketones. Different yeasts, such as brewer’s yeast, distilling yeast, wine yeast, and non-*Saccharomyces* yeast, have been investigated for whisky production [5]. Brewer’s yeast was traditionally used for malt whisky fermentation and can achieve an elevated ethanol yield [6]. A study has compared the sensory differences among several types of brewer’s yeast, e.g., lager yeast from Japan and ale yeast from UK, and found that the dry yeasts produced a highly complex character in new-make spirit, while the ale yeast produced a more estery character [7]. As for distilling yeast, the culture properties require high alcohol production, temperature tolerance, storage stability, flavor consistency, etc. [8], and the commercial distilling yeast strains in Scotch whisky involve M and MX yeast, Pinnacle Yeast, and DistillMax Yeast [9].

As for distillation, pot distillation and tower distillation are commonly used to achieve a desired alcohol strength and intense flavor. A range of 36–50% alcohol by volume (ABV) is often seen in most commercial whisky products [10]. Thus, the alcohol content of new-make spirit is usually as high as 70% ABV, considering the loss during barrel aging and the initial alcohol content for blending [10]. For this purpose, the fermented wash is generally distilled twice, producing two spirits with different ABV levels [4]. The distillates are mixed in a certain proportion from three fractions: the head, heart and tail. The head and tail fractions, which have undesirable odors, are often discarded or redistilled, while the heart, being the cleanest and most balanced part with high levels of medium-chain esters (between C6 and C12), is retained after distillation [11,12]. Therefore, through fermentation and distillation, high alcohol content and rich aroma compounds can be achieved, and choosing the appropriate fermentation and distillation process parameters is the key to the final flavor quality of whisky.

In respect of the aroma compounds determination in whisky, some combinations of sample preparation and detection methods have been applied. Common sample preparation methods include solid phase microextraction (SPME), liquid–liquid extraction (LLE), and direct injection (DI) [13]. SPME is a fast, automated, solvent-free method, which possesses high sensitivity and reproducibility [14]; however, for high-ABV alcohol samples, dilution with water is a commonly used strategy to extract more compounds with low contents. Common sample detection methods include gas chromatography–mass spectrometry (GC-MS), gas chromatography–olfactometry (GC-O), comprehensive two-dimensional gas chromatography (GC×GC), ion mobility spectrometry (IMS), electronic nose, and their combinations [15,16,17,18]. GC-MS undoubtedly is the most convenient method, but it only provides quantitative information rather than sensory information. GC×GC-O-MS offers many advantages, e.g., determining all the volatile compounds through two-dimensional separation, and confirming the odor activity of each compound [19]. The application of complementary methods can better investigate the effects of different production processes on aroma compounds. For the products, it is more worthy of attention which volatile compounds contribute significantly to the odor, according to the odor activity value (OAV) [20] or relative odor activity value (rOAV) [21].

Fermentation temperature significantly affects yeast metabolism and subsequent flavor formation. The plasma membrane is the primary site of temperature perception; low fermentation temperatures restrict yeast growth and alter membrane fatty acid unsaturation to maintain optimal fluidity [22]. Under heat stress, Saccharomyces cerevisiae accumulates trehalose and glycogen to protect cellular proteins against denaturation, while the tricarboxylic acid (TCA) cycle is enhanced to meet increased energy demands [23]. At the enzymatic level, temperature modulates the activity of key enzymes involved in aroma formation, such as alcohol acetyltransferases (Atf1 and Atf2) for ester synthesis [24]. Higher fermentation temperatures generally accelerate yeast metabolism, but may also accelerate enzyme denaturation, whereas lower temperatures promote greater ester stability and prolonged fermentation [25]. Temperature also affects the kinetics of amino acid uptake, which directly determines the availability of precursors for higher alcohol synthesis via the Ehrlich pathway [26]. Understanding these temperature-dependent metabolic shifts is therefore critical for optimizing the flavor profile of new-make whisky. However, systematic comparisons of different distilling yeasts at different fermentation temperatures under pilot-scale whisky production conditions remain limited. Specifically, no previous study has compared two distilling yeasts at two different temperatures (30 °C and 35 °C) across all four production stages (wort, wash, low wine, new-make spirit) in a single experimental setup. In this paper, the influence of fermentation and distillation on the aroma compounds in new-make whisky was investigated. Four groups of fermentation conditions were compared: distilling yeast 1 at 30 °C, distilling yeast 1 at 35 °C, distilling yeast 2 at 30 °C, and distilling yeast 2 at 35 °C. The wort and wash samples were initially determined by HPLC and UPLC-MS for the non-volatile precursors and by GC-MS for the main aroma compounds. Then, the samples with the best fermentation conditions, as well as the control samples fermented by brewer’s yeast, were analyzed by GC×GC-O-MS based on the aroma compounds in different stages of new-make whiskies. Finally, the feature aroma-active compounds in different new-make whiskies were identified according to the relative odor activity value. The novelty of this study lies in three aspects: (1) the first systematic comparison of two distilling yeasts at two fermentation temperatures; (2) the application of GC×GC-O-MS across all four production stages; and (3) the identification of yeast-specific potential aroma markers using rOAV.

## 2. Results and Discussion

### 2.1. Variation in Non-Volatile Compounds in Wash Under Different Fermentation Conditions

**Figure 1:**
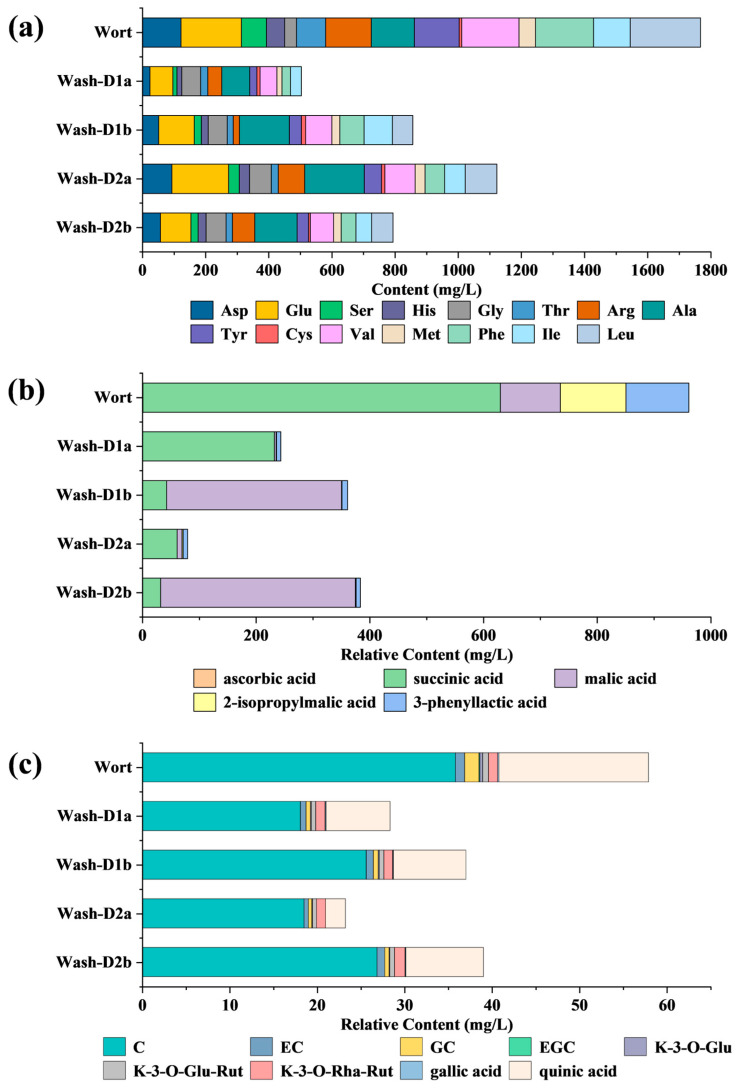
Variation and difference in (**a**) amino acids, (**b**) organic acids and (**c**) phenolic compounds in wort and wash samples through different fermentation conditions (D1a—distilling yeast 1 at 30 °C; D1b—distilling yeast 1 at 35 °C; D2a—distilling yeast 2 at 30 °C; D2b—distilling yeast 2 at 35 °C).

To ensure the best fermentation condition of two distilling yeasts, two fermentation temperatures (i.e., 30 °C and 35 °C) were selected, and the other fermentation conditions were the same. In Figure 1a, a total of 15 amino acids were detected in all wort and wash samples by HPLC. For most amino acids after fermentation, the contents were decreased, except for glycine and alanine. Some amino acids had a significant decline in content, such as serine, threonine, tyrosine, valine, phenylalanine and leucine. For example, serine decreased from 79.30 mg/L in wort to 13.29 mg/L in Wash-D1a (30 °C) and 22.72 mg/L in Wash-D2b (35 °C); threonine dropped from 91.96 mg/L to 22.86 mg/L and 20.51 mg/L, respectively. There were also some differences between different wash samples. Two distilling yeasts showed opposite results under two fermentation temperatures of 30 °C and 35 °C: for most amino acids, the wash sample fermented by distilling yeast 1 had a lower content at 30 °C (Wash-D1a), while the wash sample fermented by distilling yeast 2 had a lower content at 35 °C (Wash-D2b), which meant that distilling yeast 1 preferred consuming amino acids at 30 °C and distilling yeast 2 preferred consuming amino acids at 35 °C. This strain-dependent and temperature-dependent amino acid consumption pattern has been confirmed by recent studies. In one systematic investigation of six Saccharomyces cerevisiae strains at 20 °C, 30 °C, and 35 °C during huangjiu fermentation, it was shown that some strains consumed more amino acids at lower temperatures while others exhibited greater uptake at higher temperatures, indicating that the optimal temperature for amino acid utilization is strain-dependent [27]. Similarly, a study on nitrogen source utilization by three commercial whisky strains during Scotch grain whisky fermentation observed significant variability in nitrogen uptake among different yeast strains [28]. Our findings extend these observations to distilling yeasts in whisky production, confirming that distilling yeast 1 (preferring 30 °C) and distilling yeast 2 (preferring 35 °C) possess distinct temperature optima for amino acid consumption. It should be noted that the amino acids discussed above originate from the saccharification step, where proteins and starches in the raw materials are hydrolyzed into low molecular weight amino acids and sugars in the wort, thereby providing the essential nutrients for subsequent yeast growth and amino acid consumption during fermentation [29].

In terms of organic acids and phenolic compounds, the changes in them were different from that of amino acids. Succinic acid and malic acid, as the main organic acids, were greatly affected by temperature; succinic acid showed a significant decline in content after fermentation, especially under high temperature, while malic acid showed opposite trends at different temperatures; the contents of other organic acids were also decreased in wash samples. The determined phenolic compounds included catechins, flavonol glycosides, and phenolic acids; catechin (C) had the highest content among all the phenolic compounds in both wort and wash samples, and had a higher reduction under lower temperature after fermentation. Quinic acid also accounted for a large proportion, and showed the same trend as catechin; specifically, catechin decreased from 35.83 mg/L in wort to 18.08 mg/L in Wash-D1a (30 °C) and 26.84 mg/L in Wash-D2b (35 °C), while quinic acid dropped from 17.1 mg/L to 7.32 mg/L and 8.89 mg/L, respectively. The contents of other phenolic compounds were also decreased in wash samples. Succinic acid decreases as yeast reabsorbs it late in fermentation for the TCA cycle and amino acid synthesis, and also converts it with ethanol to diethyl succinate, which adds a faint fruity aroma [30]. Malic acid, however, responds differently to temperature: it accumulates at 35 °C under heat stress but is consumed at 30 °C due to efficient metabolic coupling [31]. Similarly, catechin and quinic acid levels drop after fermentation, mainly through protein binding and adsorption onto yeast cells [32]. The underlying mechanisms for the increased malic acid and reduced degradation of catechin and quinic acid at 35 °C are not yet fully understood. It is hypothesized that under heat stress, yeast metabolism may redirect TCA cycle flux, leading to transient malic acid accumulation, and that temperature-induced changes in cell wall composition may reduce phenolic adsorption. A similar temperature-dependent accumulation of malic acid has been reported in wine fermentations at 35 °C, which is consistent with our hypothesis that heat stress may transiently elevate TCA cycle intermediates [31]. Further targeted studies are needed to confirm these hypotheses.

### 2.2. Influence of Fermentation Conditions on the Main Aroma Compounds in Wash

**Figure 2:**
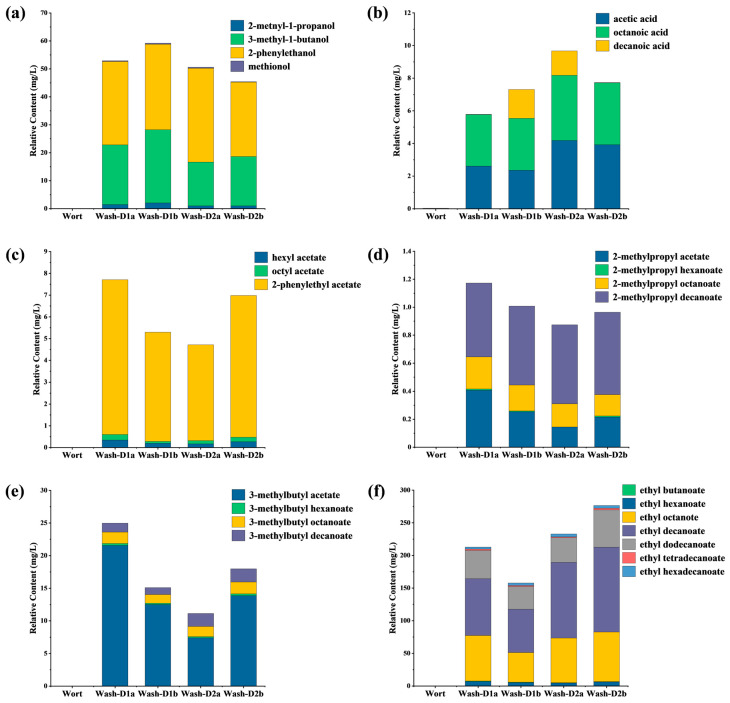
Comparison of the relative contents of main aroma compounds ((**a**) alcohols, (**b**) acids, (**c**) acetic esters, (**d**) 2-methylpropyl esters, (**e**) 3-methylbutyl esters, and (**f**) ethyl esters) in wort and wash samples through different fermentation conditions (D1a—distilling yeast 1 at 30 °C; D1b—distilling yeast 1 at 35 °C; D2a—distilling yeast 2 at 30 °C; D2b—distilling yeast 2 at 35 °C).

To obtain better fermentation conditions, the main aroma compounds in wort and wash samples in Section 3.2 were determined by GC-MS [3,14]. A total of 34 volatile compounds were identified and quantified, including eight alcohols, five acids, and 21 esters (comprising six acetic esters, five ethyl esters, four 2-methylpropyl esters, and six 3-methylbutyl esters). It was obvious that the contents of most compounds increased after fermentation in Figure 2a–f. According to the molecular structure, the aroma compounds can be separated into different categories, such as alcohols, acids, and esters. Similarly to amino acids, two distilling yeasts also showed opposite results under two fermentation temperatures of 30 °C and 35 °C. However, the results of different aroma compounds were not the same: for the alcohols (e.g., 2-phenylethanol) and acids (e.g., acetic acid), the fermentation conditions of Wash-D1b and Wash-D2a performed better than the other two, as shown in Figure 2a,b, while for the different sub-categories of esters (i.e., acetic esters, ethyl esters, 2-methylpropyl esters, and 3-methylbutyl esters), Wash-D1a and Wash-D2b obtained better results, as shown in Figure 2c–f.

Through the Strecker degradation pathway, amino acids can react with carbonyl compounds to form distinctive Strecker aldehydes [33]. Theoretically, each amino acid has its corresponding Strecker aldehydes. However, only some amino acids (i.e., glycine, alanine, valine, leucine, isoleucine, phenylalanine, and methionine) were verified as having their Strecker aldehydes (i.e., formaldehyde, acetaldehyde, 2-methylpropanal, 3-methylbutanal, 2-methylbutanal, phenylacetaldehyde, and methional) [34,35]. Aldehydes can be sequentially reduced into alcohols [36,37]. Four higher alcohols of Strecker aldehydes were determined in wash samples, i.e., 2-methyl-1-propanol, 3-methyl-1-butanol, 2-phenylethanol, and methionol, as shown in Figure 2a. As precursors, these compounds continued to undergo dehydration condensation with the acids to form esters by microbial synthesis, as seen in Figure 2c–e. Due to the high content of ethanol, the esterification reaction via ethanol and acids can be achieved, which is the reason for the high concentrations and large variety of ethyl esters after fermentation, as shown in Figure 2f. Although ethanol is also an oxidative product of Strecker aldehydes, the main formation pathway is via synthesis from glucose, xylose, and arabinose under anaerobic conditions by yeasts [38].

The above metabolic changes can also explain the production of aroma compounds and the influence of fermentation conditions on these compounds. Under the optimal conditions for yeast, more acids and alcohols are converted into esters [22,25]. Compared with previous whisky fermentation studies [3], our observed ester concentrations fall within similar ranges, confirming the general ester-forming capacity of Saccharomyces cerevisiae. However, the temperature-dependent opposite preferences of the two distilling yeasts have not been systematically reported before; this finding extends current knowledge by revealing strain-specific optimal windows for ester synthesis. Therefore, based on the results of the amino acids and main aroma compounds, the final optimal fermentation temperature for distilling yeast 1 was determined to be 30 °C, and for distilling yeast 2, it was 35 °C.

### 2.3. Exploration of Potential Metabolites in Different Stages of Whisky

**Figure 3:**
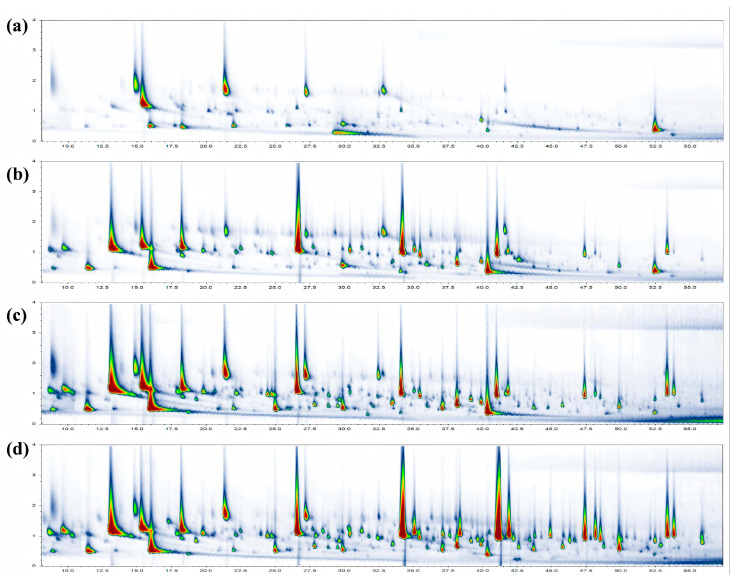
Two-dimensional chromatogram obtained via GC×GC-O-MS of different stages ((**a**) wort, (**b**) wash, (**c**) low wine, and (**d**) new-make spirit) of whisky fermented by distilling yeast 1.

Main aroma compounds before and after fermentation have been determined by GC-MS. However, more compounds with low contents need to be found, because the distillation process may increase their concentrations. Therefore, the selected wash samples (Wash-D1a and Wash-D2b) and their distilled samples (LW-D1a, LW-D2b, Spirit-D1a and Spirit-D2b), as well as the control samples (Wash-B, LW-B, and Spirit-B), were further analyzed by GC×GC-O-MS. The qualitative detection results are shown in Table 1. There were 74 compounds determined in different stages of whiskies fermented by three yeasts. More fatty alcohols (e.g., 1-hexanol and 1-octanol), fatty esters (e.g., ethyl heptanoate, and ethyl nonanoate), lactones (e.g., γ-nonalactone), fatty aldehydes (e.g., nonanal, and decanal), terpene alcohols (e.g., linalool), aromatic aldehydes (e.g., benzaldehyde), terpene aldehydes (e.g., (E)-2-nonenal) and other compounds (e.g., furfural) were found by GC×GC-O-MS than by GC-MS. This phenomenon can be explained according to the two-dimensional chromatograms in Figure 3 and Figure S1. Many of the compounds had low contents before distillation, so they may be below the limit of detection of GC-MS, or disturbed by the background or matrix. When two-dimensional GC was employed, the aroma compounds were well-separated for qualitative analysis. In addition, once the olfactory method was used, the aroma-active compounds could be easily distinguished.

### 2.4. Influence of Distillation Conditions on the Aroma Compounds in Wash

**Figure 4:**
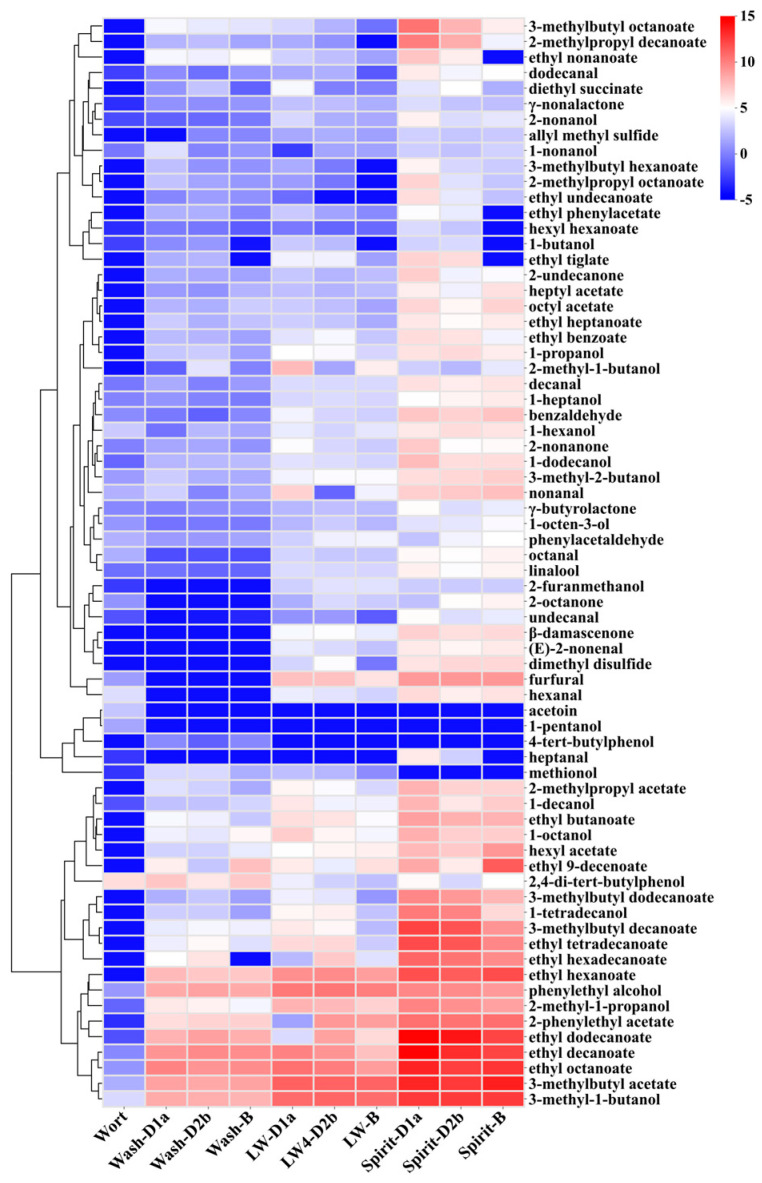
Heatmap visualization of relative contents of odor compounds in different stages (wort, wash, low wine, and new-make spirit) of whiskies fermented by different yeasts (distilling yeast 1, distilling yeast 2, and brewer’s yeast). The data was scaled by log_2_.

To better explain the changes in aroma compounds in the distillation process, the samples in different stages of whisky fermented by brewer’s yeast were compared, as shown in Figure 3 and Figure 4. After the first distillation process, the contents of most compounds in the three low wine samples were increased, and that continued in the new-make spirits after the second distillation. The concentration ratio reached 10–100 times. The representative compounds included most fatty alcohols, most fatty esters, most fatty aldehydes, 2-methyl-1-propanol, 3-methyl-1-butanol, 3-methyl-2-butanol, linalool, ethyl benzoate, and β-damascenone, amongst others. This phenomenon can be explained by the fact that these compounds had medium boiling point and good thermostability [43]. Similar enrichment factors (10- to 100-fold) have been reported for pot-still distillation of Scotch malt whisky [4], indicating that our results are consistent with previous observations. However, the complete tracking of all four production stages (wort, wash, low wine, new-make spirit) in a single study is rarely performed; this provides a more comprehensive view of aroma compound evolution than earlier reports that focused on only one or two stages. There were also a few compounds demonstrating an opposite trend, such as methionol, furfural, 2,4-di-tert-butylphenol, and allyl methyl sulfide, which were not stable in distillation. The decrease in sulfur compounds (e.g., methionol, allyl methyl sulfide) is attributed to interaction with the copper still. Cu(I) and Cu(II) form stable complexes with H_2_S and thiols, with Cu(II)-sulfide bonding energies exceeding those of Cu(I)-sulfide [44]. Under hydroalcoholic conditions, Cu(II) rapidly forms ~1.4:1 H_2_S/Cu and ~2:1 thiol/Cu complexes, oxidizing sulfur compounds and reducing Cu(II) to Cu(I), which then reacts with oxygen [45]. In whisky production, copper pot stills lower sulfuric aromas by reducing compounds like dimethyl trisulfide (DMTS) [43]. Moreover, DMDS and DMTS are produced in larger amounts in the presence of copper, from methanethiol (DMDS) or methanethiol plus H_2_S (DMTS) [10,43]. Therefore, the net effect of copper on sulfur compounds is compound-specific: some sulfur compounds (e.g., methionol, allyl methyl sulfide) are removed via complexation and oxidation, while others (e.g., DMDS, DMTS) can be formed from suitable precursors under the same conditions. The trends of phenylacetaldehyde, 2-phenylethanol, and 1-methylnaphthalene were unique: they increased first and then decreased. Interestingly, 2-methyl-1-butanol, the reduction product of 2-methylbutanal (Strecker aldehyde) that cannot be separated from 3-methyl-1-butanol by one-dimensional gas chromatography, was found by two-dimensional gas chromatography [46].

In terms of different samples fermented by three yeasts, distilling yeast 1 produced the highest contents of most aroma compounds during different stages; distilling yeast 2 produced the second and the brewer’s yeast produced the least, as shown in Figure 4. Some aroma compounds were unique: 2-octanone was not found in distilling yeast 1-fermented low wine and spirit; in brewer’s yeast-fermented wash, low wine and spirit, the compounds of ethyl tiglate, 1-butanol, and diethyl succinate were not or hardly found, while ethyl 9-decenoate had the highest content.

### 2.5. Recognition of Feature Aroma-Active Compounds in Different New-Make Malt Whiskies

Three new-make spirits produced by different yeasts (distilling yeast 1, distilling yeast 2, and brewer’s yeast) were compared. The feature aroma-active compounds of each sample were identified based on rOAV in Table 2. The odor thresholds are listed in Table 1.

The three most prominent aroma-active compounds—β-damascenone, 3-methylbutyl acetate, and 3-methylbutyl octanoate—are formed through distinct metabolic routes. β-Damascenone is a norisoprenoid derived from carotenoid degradation (e.g., neoxanthin) in barley malt. During mashing and fermentation, carotenoids are cleaved to allenic triol intermediates, which undergo acid-catalyzed rearrangement to β-damascenone [47,48]. Its extremely low odor threshold (0.002 ppb) explains its potent sensory impact at trace levels. 3-Methylbutyl acetate is synthesized by yeast via the Ehrlich pathway: leucine → α-ketoisocaproate → isoamyl aldehyde → isoamyl alcohol, followed by esterification with acetyl–CoA catalyzed by alcohol acetyltransferases (ATF1, ATF2) [49]. 3-Methylbutyl octanoate is formed from the same isoamyl alcohol, but with octanoyl–CoA as the acyl donor. The availability of octanoyl–CoA depends on yeast biosynthesis of medium-chain fatty acids, which is influenced by fermentation temperature and strain [50].

Although many compounds were detected, only those with rOAV > 1 are considered potential key odorants—mainly esters, alcohols, aldehydes, and ketones. β-Damascenone and dimethyl disulfide had relatively high rOAVs, with β-damascenone, 3-methylbutyl acetate, and dimethyl sulfide exhibiting the highest levels. β-Damascenone is a known key odorant in Bourbon whisky [42], and its extremely high rOAV (>1000) in our samples is consistent with this. Similarly, 3-methylbutyl acetate has been reported to have a high OAV in malt whisky [3], and its elevated rOAV aligns with those findings. These agreements support the reliability of our rOAV-based relative comparisons under identical analytical conditions.

A comparison between distilling yeasts and brewer’s yeast showed that distilling yeasts generally gave higher rOAVs for distinct odor compounds, indicating stronger aroma intensity. Further comparison between the two distilling yeast strains revealed that distilling yeast 1 produced a stronger aroma intensity than distilling yeast 2. The observed differences can be attributed to distinct temperature preferences and metabolic capacities. Distilling yeast 1, with its optimal fermentation temperature at 30 °C, produced higher rOAVs for fruity esters (e.g., ethyl octanoate, ethyl dodecanoate), consistent with its higher ester-forming activity under this condition. In contrast, brewer’s yeast fermented at 15 °C favored the formation of 1-octen-3-ol (mushroom-like, earthy), suggesting a shift toward different aroma pathways. Thus, yeast selection and fermentation temperature directly modulate the relative odor activity profile, providing a practical reference for tailoring whisky flavor.

## 3. Materials and Methods

### 3.1. Reagents and Materials

The reference substances of amino acids (1 nmol/μL in 0.1 M HCl), borate buffer 0.4 mol/L in water, 9-fluorenylmethylchloroformate in acetonitrile (FMOC reagent), and o-phthalaldehyde and 3-mercaptopropionic acid in borate buffer (OPA reagent) were purchased from Agilent (Santa Clara, CA, USA). The reference substances, including AR-grade 1-octanol, 1-octen-3-ol, linalool, ethyl butanoate, ethyl hexanoate, ethyl octanoate, ethyl decanoate, ethyl dodecanoate, ethyl tetradecanoate, ethyl hexadecanoate, 3-methylbutyl acetate, 3-methylbutyl octanoate, 3-methylbutyl decanoate, 2-phenylethyl acetate, hexanal, octanal, nonanal, decanal, dodecanal, (E)-2-nonenal, 2-undecanone, β-damascenone and dimethyl disulfide were purchased from Yuanye (Shanghai, China) and Aladdin (Shanghai, China). AR-grade organic acids (succinic acid, malic acid, citric acid, tartaric acid, lactic acid), phenolic compounds (catechin (C), epicatechin (EC), quinic acid, gallic acid, chlorogenic acid, quercetin-3-glucoside, kaempferol-3-glucoside), and the internal standard ethyl gallate (purity ≥ 98%) were purchased from Yuanye (Shanghai, China). AR-grade sodium chloride was purchased from Sinopharm Chemical Reagent (Shanghai, China). AR-grade 2-methyl-3-heptanone, used as the internal standard (IS) for both GC-MS and GC×GC-O-MS analysis, was purchased from Yuanye (Shanghai, China). AR-grade ethyl gallate as the IS for UPLC-MS analysis was purchased from Shanghai Zzstandard (Shanghai, China). The n-alkanes (C8-C25), used for calculating the retention indices, were purchased from Sigma-Aldrich (St. Louis, MO, USA). HPLC-grade hexane, acetonitrile, methanol, sodium phosphate monobasic dihydrate and formic acid were purchased from MREDA Technology (Beijing, China). The water was prepared by an ultrapure water machine (Zhongyang Yongkang, Beijing, China).

### 3.2. Different Stages of Whisky Samples

The same batch of 100% malted barley was brewed to produce the wort. Next, the wort sample was fermented under four groups of fermentation conditions (by two distilling yeasts at two fermentation temperatures). The fermentation temperatures of 30 °C and 35 °C were chosen for two reasons. First, distilling yeasts typically have higher optimal fermentation temperatures than brewer’s yeast. Second, these two temperatures were selected based on our previous research experience. After 3–5 days, the wash finally reached 8–10% ABV. Subsequently, the wash sample underwent the first distillation to reach approximately 25% ABV (low wine) by a copper pot still, and was then redistilled by the same apparatus until reaching ca. 70% ABV (new-make spirit) as the new-make whisky. Control samples (T5) were the different stages of whisky fermented using brewer’s yeast. Samples at the four stages of whisky production under the five fermentation conditions were collected promptly and stored at −80 °C before use. All the information on the different samples is listed in Table 3.

Note: pH and total titratable acidity (TTA) were not determined in this study. The experimental focus was placed on the profiling of volatile aroma compounds (by GC-MS and GC×GC-O-MS) and their non-volatile precursors (amino acids, organic acids, and phenolic compounds by HPLC and UPLC-MS). Therefore, pH and TTA data are not available.

### 3.3. High Performance Liquid Chromatography

First, 1.0 mL of each wort or wash sample was passed through a 0.22 μm membrane filter. Free amino acids in the samples were determined by high performance liquid chromatography (HPLC, Agilent 1200, Santa Clara, CA, USA)) through an OPA-FMOC pre-column derivatization method [21]. The separation was performed on a Zorbax Eclipse-AAA column (4.6 mm × 150 mm × 5 μm) maintained at 35 °C. Mobile phase A consisted of 40 mmol/L NaH_2_PO_4_, and was adjusted to pH 7.8 with 10 mol/L NaOH solution. Mobile phase B was a mixture of acetonitrile/methanol/water (45:45:10, *v*/*v*/*v*). Amino acid reference solutions were diluted with 0.1 mol/L hydrochloric acid water solution into different concentrations (0.0625, 0.125, 0.25, 0.5, and 1 mmol/L) for the construction of calibration curves. The flow rate was set at 1.0 mL/min, and detection was carried out by a diode array detector (DAD) at a wavelength of 254 nm.

### 3.4. Ultra-High Performance Liquid Chromatography-Mass Spectrometry

Chromatographic analysis was performed on an U3000 ultra-high performance liquid chromatography system (Thermo Fisher Scientific, Waltham, MA, USA) equipped with an Acquity UPLC BEH C18 column (150 mm × 2.1 mm, 1.7 μm, Waters, Milford, MA, USA). The autosampler temperature was set at 4 °C, the column temperature at 38 °C, and the injection volume was 2 μL. Mobile phase A consisted of 0.2% (*v*/*v*) formic acid in acetonitrile, and mobile phase B consisted of 0.2% (*v*/*v*) formic acid in water. The gradient elution program was as follows: 0–14 min, increased linearly from 5% to 25%; 14–16 min, from 25% to 98%; 16–18 min, held at 98%; 18–18.1 min, decreased from 98% to 5%; 18.1–22 min, held at 5%. The flow rate was 0.3 mL/min.

Mass spectrometry was carried out on a Q-Exactive mass spectrometer (Thermo Fisher Scientific, Waltham, MA, USA) equipped with an electrospray ionization (ESI) source operating in negative ion mode. The ion source temperature was 300 °C. The scan mode was Full MS/dd-MS^2^ over a mass range of *m*/*z* 100–1500, with a resolution of 70,000 for Full MS and 17,500 for dd-MS^2^. High-energy collisional dissociation (HCD) was used with stepped normalized collision energies of 10, 25, and 40 eV. The automatic gain control (AGC) target was set at 1 × 10^5^.

The target analytes were the organic acids and phenolic compounds listed in Section 3.1. Semi-quantification was performed using the internal standard method with ethyl gallate (500 mg/L) as the IS, following this calculation: relative content = (peak area of analyte/peak area of IS) × concentration of IS.

### 3.5. Gas Chromatography–Mass Spectrometry

Sample extraction: 4.0 mL of each wort or wash sample was loaded into a 20 mL odorless headspace vial, and added to 5.0 μL of 2-methyl-3-heptanone (0.816 μg/μL). The solid-phase microextraction (SPME) fiber assembly divinylbenzene/carboxen/polydimethylsiloxane (DVB/CAR/PDMS, 50/30 μm, 1 cm, Supelco, Bellefonte, PA, USA) was used for aroma extraction by a SPME autosampler (Gerstel, Muelheim, Germany). After incubation at 55 °C for 10 min, the aroma compounds in the liquid sample were extracted by SPME for 40 min in the headspace vial. Then, the fiber was desorbed into the GC injection port at 250 °C for 5 min.

Instrument detection: The determination of aroma compounds was performed on a gas chromatography–mass spectrometry (GC-MS) system (7890A, Agilent Technologies, Santa Clara, CA, USA). Volatile compounds were separated by a VF-WAX column (30 m × 0.25 mm × 0.25 μm, Agilent Technologies). The initial GC column temperature was set to 40 °C for 3 min; subsequently, the temperature was increased to 200 °C at a rate of 5 °C/min, and then increased to 230 °C at a rate of 10 °C/min; finally, it was held at 230 °C for 5 min, before a post run of 250 °C for 2.5 min. The flow rate of the carrier gas (helium, 99.999%) was 1 mL/min in the splitless mode. The electron energy of the EI source was 70 eV, and the scan range was *m*/*z* 40–350.

Qualitative and quantitative analysis: The aroma compounds in the liquid sample were identified through gas chromatogram and mass spectrum. The retention index (RI) values of aroma compounds were calculated according to the n-alkanes (C7-C30). The fragment ions of aroma compounds in MS were searched in the 2017 NIST library. The relative contents of aroma compounds in the liquid sample were calculated by multiplying the content of IS by the peak area ratio of the aroma compound to the IS.

### 3.6. Two-Dimensional Gas Chromatography–Olfactory–Mass Spectrometry

Sample extraction: The low wine and spirit samples were diluted with water (Millipore, Bedford, MA, USA) into 10% ABV solution before determination, and the wort and wash samples were not diluted. Then, 4.0 mL of each liquid sample was loaded into a 20 mL odorless headspace vial, and 5.0 μL of 2-methyl-3-heptanone (0.816 μg/μL) and saturated sodium chloride were also added. After incubation at 55 °C for 10 min, the aroma compounds in the liquid sample were extracted by SPME (DVB/CAR/PDMS, 50/30 μm, 1 cm, Supelco, Bellefonte, PA, USA) for 40 min in the headspace vial. Then, the fiber was desorbed into the GC injection port at 250 °C for 5 min.

Instrument detection: The determination of the aroma compounds was performed on a two-dimensional gas chromatography–olfactometry–mass spectrometry (GC×GC-O-MS) system (8890, Agilent Technologies, Santa Clara, CA, USA), equipped with a Sniffer 9100 sniffing-port (Brechbühler, Schlieren, Switzerland). Volatile compounds were sequentially separated by two columns, i.e., DB-WAX (30 m × 0.25 mm × 0.25 μm, J&W Scientific, Folsom, CA, USA) and DB-17 (1.85 m × 0.18 mm × 0.18 μm, J&W Scientific). The two columns were controlled by an SSM1800 solid modulator (J&W Scientific, Folsom, CA, USA). The initial GC column temperature was set to 40 °C for 3 min; subsequently, the temperature was increased to 200 °C at a rate of 5 °C/min, and then increased to 230 °C at a rate of 10 °C/min; finally, it was held at 230 °C for 5 min, before a post run of 250 °C for 2.5 min. The flow rate of the carrier gas (helium, 99.999%) was 1 mL/min in the splitless mode. The electron energy of the EI source was 70 eV, and the scan range was *m*/*z* 40–350.

The GC-O analysis was performed by a sensory panel consisting of three trained assessors (one male, two females, aged 20–25 years). All assessors had at least 100 h of prior GC-O experience and were trained for an additional 20 h using 20 standard odorants (e.g., isoamyl acetate, β-damascenone, dimethyl disulfide) at various concentrations. During GC-O, each assessor recorded the odor description and intensity in real time using a sniffing port (Sniffer 9100). A compound was considered olfactorily detected if at least two out of three assessors perceived an odor at the same retention time.

Qualitative and quantitative analysis: The aroma compounds in the liquid sample were identified through gas chromatogram, mass spectrum and sniffing results. The retention index (RI) values of the aroma compounds were calculated according to the n-alkanes (C7-C30). The fragment ions of aroma compounds in MS were searched in the 2017 NIST library. The aroma descriptions of the analytes were recorded in real time by the sensory panel, and searched in the flavor library (www.perflavory.com). The relative contents of the aroma compounds in the liquid sample were calculated by multiplying the content of IS by the peak area ratio of the aroma compound to the IS. The rOAVs of aroma compounds in the liquid sample were the ratios of the relative content to the corresponding odor threshold.

### 3.7. Statistical Analysis

All experiments were performed in triplicate (three technical replicates). No biological replicates were performed due to the pilot-scale nature of the work. Data are presented as mean values. The relative contents of aroma compounds were calculated using the internal standard method, as described in Section 3.5 and Section 3.6.

## 4. Conclusions

In this study, the effects of fermentation and distillation on the aroma of new-make whisky were revealed. When comparing different fermentation conditions (i.e., two distilling yeasts and two fermentation temperatures), distilling yeast 1 at 30 °C and distilling yeast 2 at 35 °C produced better results. The contents of the main aroma compounds, such as 2-phenylethanol and acetic acid, increased, while the contents of amino acids decreased after fermentation, as determined by GC-MS and HPLC, respectively. The metabolic pathways were summarized as Strecker degradation, oxidation, and esterification. After two distillations, more compounds were identified by GC×GC-O-MS, in particular, 2-methyl-1-butanol, which cannot be separated from 3-methyl-1-butanol by GC-MS. Most of the compounds showed up to a 100-fold increase in content, while methionol, furfural, 2,4-di-tert-butylphenol, and allyl methyl sulfide showed decreased content. Finally, the reduction in some sulfur compounds may be due to the interaction with the copper still. In the end, feature aroma-active compounds with rOAV > 1 were confirmed in three new-make whiskies fermented by three yeasts, and there were some differences among them, such as 2-octanone, ethyl tiglate, 1-butanol, and diethyl succinate. pH and total titratable acidity data are not available, as the study focused on aroma compounds and their precursors.

In summary, this study confirms the general pathways of Strecker degradation, esterification, and copper–sulfur interactions reported in previous whisky research. It extends current knowledge by demonstrating, for the first time under pilot-scale conditions, that different distilling yeasts exhibit opposite optimal temperature windows for amino acid consumption and ester synthesis. Additionally, our results challenge the assumption in some one-dimensional GC studies that 2-methyl-1-butanol and 3-methyl-1-butanol can be reliably separated by retention time alone, highlighting the need for two-dimensional GC for accurate analysis. The above results provided a theoretical basis of process selection in the whisky industry, and serve as a reference strategy for selecting appropriate methods for different research questions.

## Figures and Tables

**Table 1:** Qualitative information on aroma compounds in all whisky samples determined by GC×GC-O-MS.

No.	Compounds	CAS	RI ^a^	Odor Description ^b^	Odor Threshold (μg/L) ^c^
Alcohols					
1	2-methyl-1-propanol	78-83-1	1007	malty	101,000
2	3-methyl-1-butanol	123-51-3	1114	malty	56,100
3	2-methyl-1-butanol	137-32-6	1111	alcoholic	64,844
4	3-methyl-2-butanol	598-75-4	1258	fruity	-
5	1-propanol	71-23-8	927	alcoholic	54,000 ^d^
6	1-butanol	71-36-3	1054	sweet	2730 ^d^
7	1-pentanol	71-41-0	1153	sweet	150 ^e^
8	1-hexanol	111-27-3	1253	fruity	5370 ^d^
9	1-heptanol	111-70-6	1355	musty	10,076
10	1-octanol	111-87-5	1455	waxy	29
11	1-nonanol	143-08-8	1528	fatty	324
12	1-decanol	112-30-1	1579	floral	5000 ^e^
13	1-dodecanol	112-53-8	1680	earthy	1000 ^e^
14	1-tetradecanol	112-72-1	1863	fruity	-
15	2-nonanol	628-99-9	1428	waxy	-
16	1-octen-3-ol	3391-86-4	1348	mushroom-like	6.1 ^d^
17	linalool	78-70-6	1446	floral	98
18	2-phenylethanol	60-12-8	1628	rosy	2600 ^f^
Acids					
19	2-ethylbutanoic acid	88-09-5	1506	acidic	-
20	hexanoic acid	142-62-1	1033	sour	7563 ^f^
Esters					
21	ethyl butanoate	105-54-4	957	fruity	112
22	ethyl hexanoate	123-66-0	1164	fruity	270
23	ethyl heptanoate	106-30-9	1266	fruity	971
24	ethyl octanoate	106-32-1	1367	wine	256
25	ethyl nonanoate	123-29-5	1469	wine-like	1639
26	ethyl decanoate	110-38-3	1537	wine	2972
27	ethyl undecanoate	627-90-7	1587	soapy	-
28	ethyl dodecanoate	106-33-2	1641	floral	500 ^d^
29	ethyl tetradecanoate	124-06-1	1780	sweety	4000 ^e^
30	ethyl hexadecanoate	628-97-7	1984	fruity	1000 ^e^
31	ethyl tiglate	5837-78-5	1158	fruity	-
32	diethyl succinate	123-25-1	1527	fruity	353,000 ^d^
33	ethyl 9-decenoate	67233-91-4	1556	fruity, fatty	-
34	hexyl acetate	142-92-7	1201	fruity	-
35	hexyl hexanoate	6378-65-0	1254	fruity	6400 ^e^
36	heptyl acetate	112-06-1	1300	wine	830 ^e^
37	octyl acetate	112-14-1	1403	earthy	-
38	2-methylpropyl acetate	110-19-0	920	sweet	1540
39	2-methylpropyl octanoate	5461-06-3	1493	floral	-
40	2-methylpropyl decanoate	30673-38-2	1597	wine	-
41	3-methylbutyl acetate	123-92-2	1047	banana-like	154
42	3-methylbutyl hexanoate	2198-61-0	1398	fruity	-
43	3-methylbutyl octanoate	2035-99-6	1550	fruity	70 ^e^
44	3-methylbutyl decanoate	2306-91-4	1652	fruity	5000 ^e^
45	3-methylbutyl dodecanoate	6309-51-9	1804	wine	100,000 ^e^
46	ethyl benzoate	93-89-0	1528	fruity	301
47	ethyl phenylacetate	101-97-3	1581	floral	781
48	2-phenylethyl acetate	103-45-7	1594	floral	983
49	γ-butyrolactone	96-48-0	1480	creamy	-
50	γ-nonalactone	104-61-0	1697	coconut-like	71
Aldehydes					
51	hexanal	66-25-1	1010	fatty	57
52	heptanal	111-71-7	1109	fatty	1069
53	octanal	124-13-0	1217	fatty	40
54	nonanal	124-19-6	1320	rosy	122 ^f^
55	decanal	112-31-2	1428	citrus-like	95
56	undecanal	112-44-7	1517	floral	5000 ^e^
57	dodecanal	112-54-9	1570	soapy	10 ^e^
58	benzaldehyde	100-52-7	1401	almond-like	5451
59	phenylacetaldehyde	122-78-1	1508	sweet	709
60	(E)-2-nonenal	18829-56-6	1450	fatty	4.4
Ketones					
61	2-octanone	111-13-7	1211	earthy	220 ^f^
62	2-nonanone	821-55-6	1313	sweet	1231
63	2-undecanone	112-12-9	1512	waxy	5.5 ^e^
64	2-tridecanone	593-08-8	1618	fatty	-
65	acetoin	513-86-0	1165	creamy	31,245
66	β-damascenone	23726-93-4	1600	honey-like	0.1 ^f^
Sulfur compounds					
67	methionol	505-10-2	1536	onion-like	2110
68	dimethyl disulfide	624-92-0	975	sulfurous	1.1
69	allyl methyl sulfide	10152-76-8	1506	onion-like	-
Others					
70	4-tert-butylphenol	98-54-4	1915	smoky	800 ^e^
71	2,4-di-tert-butylphenol	96-76-4	1952	phenolic	500 ^e^
72	furfural	98-01-1	1327	bread-like	7949
73	2-furanmethanol	98-00-0	1501	bread-like	15,000
74	3-ethoxy-1-propanol	111-35-3	1260	fruity	50,000

^a^ RI, retention index (calculated on the polar DB-WAX column). ^b^ The description was derived from olfactory results, relevant references, and the odor library (http://www.flavornet.org/flavornet.html (accessed on 26 August 2025)). ^c^ Odor thresholds determined in 50% ABV, taken from ref [5]. ^d^ Odor thresholds determined in 46% ABV, taken from ref [39,40]. ^e^ Odor thresholds were taken from ref [41]. ^f^ Odor thresholds determined in 40% ABV, taken from Ref. [42].

**Table 2:** Feature aroma-active compounds selected in different new-make spirits.

Aroma Compound ^a^	Relative Odor Activity Value (rOAV)		
	New-Make Spirit Produced by Distilling Yeast 1	New-Make Spirit Produced by Distilling Yeast 2	New-Make Spirit Produced by Brewer’s Yeast
1-octanol	9.9	4.2	4.3
1-octen-3-ol	2.3	2.5	4.4
ethyl butanoate	3.9	2.5	2.3
ethyl hexanoate	13	10	15
ethyl octanoate	44	22	28
ethyl decanoate	8.0	2.9	1.6
ethyl dodecanoate	53	31	9
ethyl tetradecanoate	1.0	<1	<1
ethyl hexadecanoate	2.1	1.4	<1
3-methylbutyl acetate	69	41	80
3-methylbutyl octanoate	21	3.7	<1
3-methylbutyl decanoate	1.0	<1	<1
2-phenylethyl acetate	1.6	1.4	1.6
hexanal	1.6	<1	1.2
nonanal	1.0	1.1	1.5
octanal	<1	<1	1.1
dodecanal	5.7	2.3	3.0
(E)-2-nonenal	13	9.4	13
2-undecanone	23	4.1	5.1
β-damascenone	1225	774	888
dimethyl disulfide	64	90	88

^a^ Aroma compound with rOAV > 1 in at least one sample.

**Table 3:** Information on all the samples.

No	Yeast	Temperature	Samples			
			Wort	Wash	Low Wine	Spirit
T1	distilling yeast 1	30 °C	Wort	Wash-D1a	LW-D1a	Spirit-D1a
T2	distilling yeast 1	35 °C	Wort	Wash-D1b	LW-D1b	Spirit-D1b
T3	distilling yeast 2	30 °C	Wort	Wash-D2a	LW-D2a	Spirit-D2a
T4	distilling yeast 2	35 °C	Wort	Wash-D2b	LW-D2b	Spirit-D2b
T5	brewer’s yeast	15 °C	Wort	Wash-B	LW-B	Spirit-B
Alcohol by volume			0%	8–10%	23–25%	71–72%

## Data Availability

No datasets were generated or analyzed during the current study.

## Supplementary Materials

The following supporting information can be downloaded at https://www.mdpi.com/article/10.3390/molecules31122138/s1, Figure S1: Two-dimensional chromatogram of different stages ((a) wort, (b) wash, (c) low wine, and (d) new-make spirit) of whisky fermented by distilling yeast 2 through GC×GC-O-MS; Figure S2. Two-dimensional chromatogram of different stages ((a) wort, (b) wash, (c) low wine, and (d) new-make spirit) of whisky fermented by brewer’s yeast through GC×GC-O-MS.

## Abbreviations

The following abbreviations are used in this manuscript:

ABV	alcohol by volume
AGC	automatic gain control
CAS	Chemical Abstracts Service
DAD	diode array detector
DI	direct injection
DMDS	dimethyl disulfide
DMTS	dimethyl trisulfide
DVB/CAR/PDMS	divinylbenzene/carboxen/polydimethylsiloxane
ESI	electrospray ionization
FMOC	9-fluorenylmethylchloroformate
GC	gas chromatography
GC×GC	comprehensive two-dimensional gas chromatography
GC-MS	gas chromatography–mass spectrometry
GC-O	gas chromatography–olfactometry
GC×GC-O-MS	two-dimensional gas chromatography–olfactometry–mass spectrometry
HCD	high-energy collisional dissociation
HPLC	high performance liquid chromatography
IMS	ion mobility spectrometry
IS	internal standard
LLE	liquid–liquid extraction
MS	mass spectrometry
OAV	odor activity value
OPA	o-phthalaldehyde and 3-mercaptopropionic acid in borate buffer
RI	retention index
rOAV	relative odor activity value
SPME	solid phase microextraction
TCA cycle	tricarboxylic acid cycle
UPLC	ultra-high performance liquid chromatography
UPLC-MS	ultra-high performance liquid chromatography–mass spectrometry
