# Supplementary material for: Influence of Different Fermentation Conditions on the Aroma-Active Compounds During New-Make Whisky Production Determined by GC-MS, GC×GC-O-MS, HPLC, and UPLC-MS
Source: Molecules. 2026 Jun 17;31(12):2138. doi: 10.3390/molecules31122138 (PMC13305191; doi:10.3390/molecules31122138)
Supplement: Supplementary file 1 [file molecules-31-02138-s001.zip › molecules-4307073-supplementary.pdf]

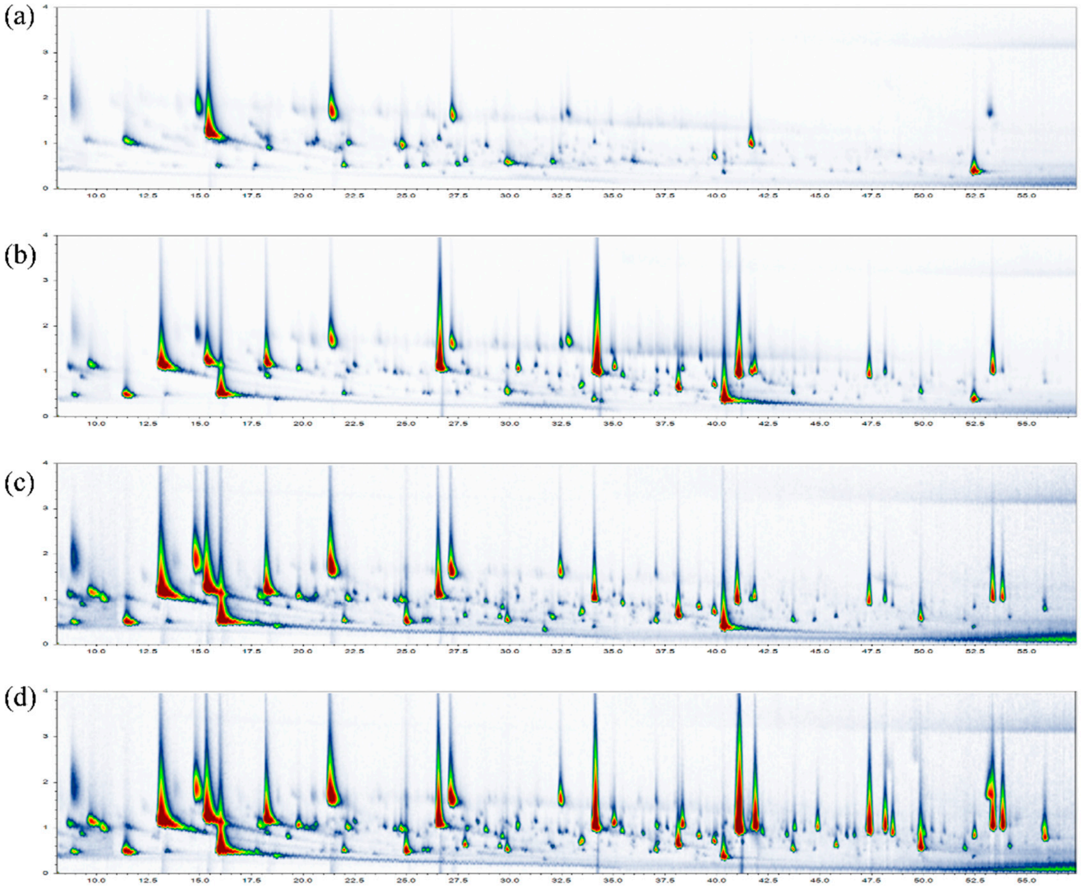

Figure S1. Two-dimensional chromatogram of different stages ((a) Wort, (b) Wash, (c) Low wine, and (d) New-make spirit) of whisky fermented by distilling yeast 2 through GC×GC-O-MS

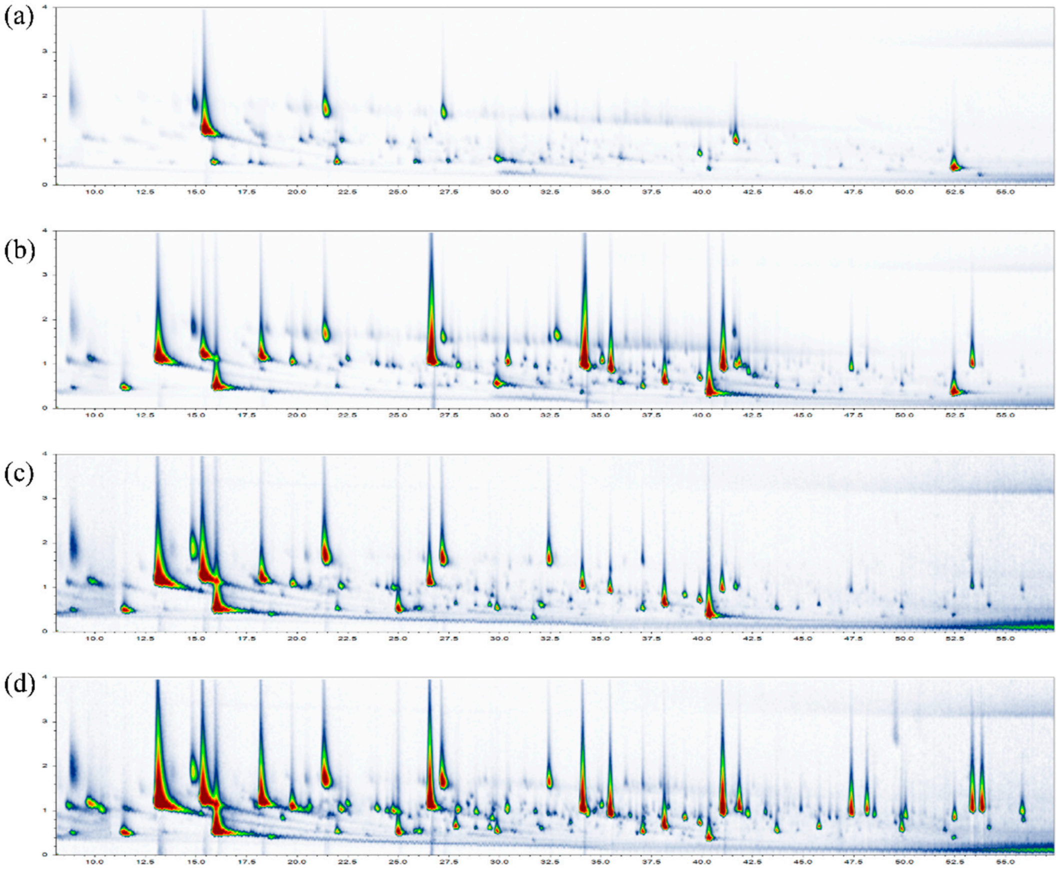

Figure S2. Two-dimensional chromatogram of different stages ((a) Wort, (b) Wash, (c) Low wine, and (d) New-make spirit) of whisky fermented by brewer's yeast through GC×GC-O-MS
